# The Complex Dynamics of Healthism in Birth Cohort Studies: Comparative Perspectives From the United Kingdom, the Netherlands, Brazil and Portugal

**DOI:** 10.1111/1467-9566.70245

**Published:** 2026-08-06

**Authors:** Sahra Gibbon, Carola Tize, Taylor Riley, Tatiane Muniz

**Affiliations:** ^1^ University College London London UK

## Abstract

Longitudinal birth cohort studies are an under‐examined context for considering the dynamics of healthism. This article presents a comparative perspective from the ‘Biosocial Lives of Birth Cohorts’ study, which takes birth cohorts as an ethnographic object of knowledge‐making, social practice and participation in the Netherlands, Brazil, Portugal and the United Kingdom. Drawing from ethnographic research with participants and professionals involved in cohort data collection, we examine how, in the routine monitoring and measuring of bodies and lives in cohort studies, ‘healthism’, as an individualised form of moral self‐responsibility for health, is entangled with medicalisation. Healthism is also at stake in the way that individual cohort test results are communicated and received. Yet the relational dynamics of cohort studies, which encompass but also exceed the scope of healthism, are also shaped by the precarity of public health infrastructures and closely entangled with the motivational desire of cohort participants, as birth cohorts can act as a stopgap for public health failures. In this way, we demonstrate how birth cohorts are an important context for considering the politics of health and how medicalisation is entangled with healthism in cohort data collection, even as this unfolds in tension with efforts to collectively intervene in public health.

## 1 Introduction

Longitudinal birth cohort studies, which follow participants and their families throughout their lives, using routine and regular collection and evaluation of social and biological data, provide an important and yet relatively under‐examined context for considering the multiple articulations and dynamics of healthism. In this article, we place birth cohorts centre stage to explore the role they play in healthism, both as an institutional research context that produces knowledge and discourses around health and lifestyles and as a social practice through participation and the relational encounter of data collection. Making use of comparative ethnographic data from our project with four regional birth cohorts—in the United Kingdom, Brazil, Portugal and the Netherlands—we examine how healthism emerges and is translated in the birth cohorts’ lifeworlds. We show how healthism is not linear or stable but, as Crawford's influential formulation emphasised, is a process “in flux”, entangled with “anxieties, hopes for health” and questions of “accountability and responsibility” (1980: 368).

Following from Crawford's definition of healthism as a moral preoccupation with health as “a goal which is to be attained primarily though the modification of life styles, with or without therapeutic help” (1980: 368), we examine how healthism and its diverse manifestations in the practices of birth cohort studies are continuously interwoven with a shared goal of constructing healthy subjects, for what are, in effect, multigenerational participants. As Crawford (1980: 368) points out, “a focus on personal health and individual lifestyle modifications may… co‐exist with and even act to stimulate attempts to change social conditions detrimental to everyone's health.” Drawing from these insights, we illuminate how healthism is a vehicle for sustaining cohort participation as a moral ‘good’ by both participants and researchers. Healthism is thereby contributing not only to individual but also to greater collective public health benefits, acting for some as a form of civic duty. We argue that these diverse tendencies, which elevate health as a ‘pan‐value’ (Turrini 2015), not only expose the multiple valences of healthism but also reflect the situated sociohistorical contexts of specific birth cohort studies, the diverse politics of access to and resources for public health in each case and the different sociocultural meanings of participation in longitudinal research.

We first examine how, in light of discussions of medicalisation and healthism, birth cohorts' scientific agendas lay the foundations for creating healthier future generations and thereby inadvertently promoting a healthism paradigm, alongside the goal of improving public health. Second, we explore how, in everyday practices within the birth cohort context, ‘healthism’ is inseparable from ‘medicalisation’ in the monitoring and measuring of bodies and lives in the routine practices of data collection including, for example, anthropometric measurements, scans and the collection of biological samples.^1^ These measurements and the numbers they produce, while potentially entailing biological reductionism, are, we demonstrate, collectively achieved, diversely desired and morally evaluated by researchers and cohort participants in ways that implicate different idioms of healthism. We then comparatively examine how formal and informal results are communicated to participants to show how both the values and responsibilities of a commitment to a wider collective investment in cohort research as public health *and* healthism are manifest and reflect the sociocultural and historic context of each cohort study.

By critically reflecting on these themes in the relatively under‐explored context of birth cohort studies, we aim to demonstrate how these unique and globally growing research communities are important sites for considering the politics of health, how medicalisation is closely tied to healthism in birth cohort data collection practices and how, for scientific communities and participants who sustain birth cohort research, neoliberal values central to healthism sit in tension with efforts to intervene in the health of populations as public health. In sum, we suggest that viewing birth cohorts through the lens of healthism enables us to better understand how research participation is a historically situated social practice made meaningful as a cultural and moral category of lived experience. This is an important step in acknowledging the present and future values of such studies and a contribution to the ongoing effort to rethink and reimagine what lifelong participation in birth cohort studies is and could be.

## 2 Methodology

Our article draws from research in four national contexts in the United Kingdom, Portugal, Brazil and the Netherlands as part of a larger project, *Biosocial Lives of Birth Cohorts.* This comparative multinational project takes the birth cohort as an object of ethnographic attention, while exploring it as a site of biosocial knowledge‐making, with a specific focus on the social practice of data collection and intergenerational participation. The four authors of this paper each carried out research in one of the cohorts: Generation 21 in Porto, Portugal; Generation R in Rotterdam, the Netherlands; the Avon Longitudinal Study of Parents and Children (ALSPAC) in Bristol, United Kingdom; and the Pelotas Birth Cohort Study in Pelotas, Brazil.

Each researcher completed, on average, 18 months of ethnographic research, carrying out interviews with participant parent–child pairs in their homes (approx. n = 30 per cohort), and individual interviews and observations with participants as they completed the routine clinical examinations and cohort evaluations that take place every 3–5 years (n = 6–30 per cohort). Each researcher also completed a photovoice project with intergenerational family pairs (n = 5–6 per cohort).^2^ All interviews were recorded, transcribed and analysed by the four authors of this article.^3^ Analysis of these data was based on a thematic approach that draws from situational analysis (A. E. Clarke et al. 2017) to identify common and comparative themes while seeking to examine these in broader historical and social contexts. We aimed to identify themes that specifically related to the intersection between how health was framed and discussed in the context of cohort evaluations and in the narrated experience of participants in the cohort studies. Research permission was granted for the entire study by University College London Research Ethics (ID: 0547/006), and local approval was sought and secured within four individual cohorts and local affiliating university institutions.^4^ Written informed consent was obtained from all participants.^5^

## 3 Situating Birth Cohort Studies in the Realm of Medicalisation and Healthism

Historically, longitudinal cohort studies have been concerned with structural factors that contribute to poor health and health disparities and have aimed at identifying and reducing risk factors for the collective benefit of wider society. The strong conflation between parenting, healthy lifestyle practices and the reduction of risk factors has made children's bodies the specific focus of anxieties regarding the health of populations (Fattore 2020). It is therefore no surprise that the establishment and creation of birth cohort studies have often been directly managed and, in part, funded within public health departments of public universities and linked to the formation and emergence of national public health systems, including in the United Kingdom (Pearson 2016) and Brazil (Victora et al. 2003). In addition, as a research method central to life course and DOHaD (developmental origins of health and disease)‐focused approaches, birth cohort studies have provided a research population and tool for articulating and examining the social determinants of health (Gibbon and Pentecost 2019; Pentecost et al. 2024; Power et al. 2013).

Despite this focus on the goals of public health, the monitoring and measuring of participants' bodies and lives might be seen as an exemplary case of medicalisation in daily life. The critique of medicalisation has been central to social science engagement with the expanding scope of biomedicine and medical authority (Conrad 1992; Zola 1972). It has been used most commonly to refer to the impact of medical terms and quantifying approaches, which are perceived as being increasingly applied to a broader range of health conditions. This is understood as both reductionist in ignoring how wider social contexts impact health and objectifying in its focus on the biological body and shifting responsibilities for health onto the individual (cf. Oakley 1985). At the same time, the potential benefits of medicalisation have also, more recently, been considered, including how the individual or collective agency of patients or publics manifests in the pursuit or result of medicalisation (Rose 2007; A. Clarke et al. 2010), thereby reflecting the entanglement between healthism and medicalisation. As we outline below, these dynamics closely intersect in the work of sustaining birth cohort studies, and, although sometimes in tension, they can also be mutually reinforced at different moments in the relational and social contexts of cohort data collection and participation.

It is significant that the ascendency of the critique of medicalisation in the 1970s and 80s coincided with both an expansion of birth cohort studies and the emergence of the conceptualisation of healthism. Although birth cohorts were initially focused on early life and social conditions, by the early 1970s, they began including a focus on preventive health behaviours such as smoking, diet, exercise and reducing alcohol intake (Wadsworth 2010; Krieger 2011). This shift to intervention reflects, in part, the expansion of a discourse of healthism and an emphasis on individual moral responsibilities for health. As Crawford states, “health talk became responsibility talk. The body's truth became an axiom of the body politic” (2006: 410). In this sense, birth cohort studies, public health and the growth of healthism have arguably propelled each other, with the former providing a resource and context for examining the effects of healthier lifestyles—and healthism—in an era of expanding neoliberal public and personal health approaches. Ultimately, the use of cohort data to track lifestyle behaviours, risk and responsibility exemplifies the very process that Crawford (1980) defines as healthism.

At the same time, it is important to note that healthism is conceptually permeable. Since Zola's (1977) inception and Crawford's influential 1980 conceptualisation, healthism has been variously defined and interpreted. For example, healthism has been applied with both positive and negative associations in research on individual health practices (e.g., Clark 2018), health technologies (e.g., Wieczorek and Rossmaier 2023), state policy and disciplinary control (e.g., Ayo 2012), and societal values, while also being variably inflected with socioeconomic and class differences (e.g., Crawford 2006). The negative applications range from, for example, framing obesity or ill health as an individual failure and ignoring structural and social causes (Jiménez‐Loaisa et al. 2020), as well as blaming mothers for moralised health choices (Fullagar 2008). Contrarily, healthism's positive associations have focused on preventive behaviours (Crawford 2006), creating activism or claiming agency over health (Turrini 2015). These multiple and diverse aspects of healthism are also evident in the evolving context of birth cohort studies and research.

In fact, birth cohort research today coincides with an increasing focus on healthism, manifested partly in the anxiety, concern and sense of felt responsibility experienced by participants over their own and their family's health; this focus, in turn, helps sustain participants’ commitment and the ongoing success of cohort studies. Participants *are* the birth cohort study in that they generate the study's data resource, which is then used by affiliated researchers to pursue broader research agendas. Because cohort studies are traditionally conceived as primarily observational rather than intervention‐focused and aimed at benefitting wider society, they rely heavily on the altruism of participants. Thereby, attrition and loss of participants to the study are a continuous challenge—and, as a result, healthism functions as both a threat and an opportunity. That is, even as birth cohort research communities seek to maintain the original goals of public health, they must respond to an increasing public health emphasis on individual health promotion while making efforts to secure the ongoing lifetime involvement of participants. This complex intersection between public health and healthism in birth cohort research is also, in part, reflected in the diverse genealogies of the cohorts we worked with.

The Generation R study, located in the Erasmus Medical Center Rotterdam,^6^ recruited 9778 mothers and partners in Rotterdam with a delivery date between April 2002 and January 2006. On‐site data collection, including scans, tests and biological sampling in children and parents, has roughly taken place every 4 years since age 5, with more frequent home questionnaires. The initiation of Generation R was directly linked to the existing infrastructure of previous cohort studies, alongside the appeal of Rotterdam's growing multiculturalism and diverse social classes. With its primary focus on women and children's health (Kooijman et al. 2016), one of the central aims of Generation R has been to better understand and improve health and equal opportunity amid rising diversity.

The ALSPAC birth cohort^7^ has been operating in Bristol since 1991 and is part of a rich legacy of birth cohort studies in the United Kingdom (Pearson 2016). Pregnant women resident in the former county of Avon, which includes the city and area around Bristol, with expected dates of delivery between 1st April 1991 and 31st December 1992 were invited to take part in the study. A total of 20,248 pregnancies were identified as eligible and 14,541 pregnancies were enrolled, leading to 13,988 children alive at 1 year of age.^8^ As an intergenerational study following families across three generations, ALSPAC also reaches out to enrol partners of the original study children who are now in their 30s. The study originally sought to focus on early childhood in a depth beyond that found in both DOHaD and the burgeoning realm of GWAS (genome‐wide association study), thereby becoming a prominent regional United Kingdom birth cohort based in Bristol with a vast local data resource.

The Generation 21 cohort is linked to the University of Porto and the Institute of Public Health (ISPUP). Established in 2005, the cohort recruited 8647 participants and their families from the public health hospitals in the metropolitan area of Porto. Although Generation 21 built on the existing infrastructure of other ISPUP longitudinal population studies, it continues to be the only birth cohort in Portugal. The study initially focused on prenatal and postnatal development and the impacts on health in later life. Participants and their families were initially assessed at 6, 15 and 24 months and then at 4, 7, 10 and 13 years, with the most recent 18‐year‐old evaluations starting in 2023. Generation 21 has generated research concerning perinatal and paediatric health with a particular focus on obesity, metabolism and cardiovascular health. It has also been part of significant European cohort consortia collaborations such as ‘Lifepath’, focused on socioeconomic differences and health outcomes.

The Pelotas Birth Cohort Study in the city of Pelotas, Southern Brazil, was initiated in 1982 by researchers at the Center of Epidemiological Research (CPE) at the Federal University of Pelotas (UFPEL) at a time of important political change when Brazil was re‐democratising and the public health system was formed. There are now 5 cohort studies in Pelotas, with the most recent starting in 2026. The 1993 cohort, and the focus of our ethnographic project, recruited all 5429 children born that year in the urban area of Pelotas. During the 30 years of follow‐up, researchers have collected a breadth of biological and social information to evaluate health indicators throughout the life course, using comparisons across the different cohorts. Findings have been especially significant for maternal health policies, with the study routinely identified as one of the most successful cohorts in a low‐to‐middle‐income country (LMIC).

These brief histories show how birth cohorts have variously emerged at the intersection of public health and efforts to address the social determinants of health. At the same time, in alignment with the expansion of health promotion, they have been concerned with widening healthcare access and participation or, in some cases, informing the promise of personalised medicine through engagement with genomics. In the next section, we draw on our ethnographic observations of the social practices of data collection to further illuminate how lifelong participation entails a process of quantification. We demonstrate how the apparent medicalisation of participants’ lives is equally synonymous with healthism.

## 4 Monitoring Health in Birth Cohort Studies

Anthropometric measurements, including height, weight, waist circumference and blood pressure measurements, are routine components of evaluations across all four birth cohorts. Ethnographic observations of the quotidian nature of anthropometric measurement provide key opportunities to understand how medicalisation and healthism are entangled in the social dynamics and relational practices of data collection.

In this section, we consider how embodied quantification, or the process of translating life experiences as data and measurements into numbers (Maturo 2015), and collecting data based on personal physical parameters, is enacted in the relational process of routine birth cohort evaluations. We examine how cohort participants and researchers navigate the ever‐present possibility of reductionism or objectification in the way lives and bodies are measured and monitored. At the same time, these socially situated forms of surveillance entail and facilitate discourses concerning the moral worth of a healthy lifestyle and participants’ own diversely stratified engagements with healthism.

In Generation 21, in Porto, evaluations for 18‐year‐olds and their mothers required physical measurements of both participants in separate clinical spaces. The anthropometric measurements are done first, with careful social choreography in the small rooms, before participants can break their overnight fast and continue with other aspects of the cohort evaluations. Participants are first asked to remove their outer clothes and are then required to move around the room, jostling with chairs, scales and tables as their height, weight, hip and waist circumference are recorded. For some, it is clearly an uneasy experience. Notably, it was often at this point in the evaluations that questions from the clinic staff (CS)^9^ about studies, university, school and holidays tumbled out. The enforced casualness of these conversations seemed to highlight the effort to ameliorate, on the part of the CS, the discomfort and potential for objectification in the work of measurement, as the technician stood in abnormal proximity to participants to obtain accurate anthropometric measurements. Yet there is also significant interest by many participants in the process of measurement itself and in the comments casually conveyed in passing by the CS.

For example, measuring body weight and composition via bioelectrical impedance analysis (BIA) was undertaken, although the participant was lying passively on the examination bed with electrodes attached to key parts of their body so that an imperceptible current could measure the water and fat content of their body. Although the process of attaching the electrodes might be imagined to be alienating or objectifying, there was little verbal protest from the young participants and often, by contrast, a sense of fascination. The CS would also, while recording the results, often make casual comments to participants. For example, observed comments by the CS to athletic male participants might be “very good” or “above average”, while making explicit a causal link between participants’ lifestyle and the numbers they read from their screen.

On one occasion, Filipe, an 18‐year‐old regular gym‐goer, after hearing about his ‘good numbers’, immediately reacted and tried to lean over to try and see Silvia's, the CS's, screen more clearly. Silvia then reached for a plastic‐covered information sheet that laid out clearly the ‘normal’ parameters for body mass index (BMI) and blood pressure testing. “You are here” she said, pointing to the above‐average quartile of the chart, adding again, “it's a really good result.” Filipe seemed pleased and notably more relaxed for the rest of the physical examination.

Lung function testing is another common measurement in birth cohort studies, and in Generation 21, it is done routinely. It is typically measured using a spirometer to assess the amount of air that can be held and how forcefully it can be expelled from the lungs. Although other cohort measurements are seemingly passive, the lung function test requires active bodily efforts on behalf of participants *and* the researcher, as one Generation 21 spirometry test below illustrates:

Jose, aged 18, enters the small lung testing room dominated by equipment. A tube with a mouthpiece is aligned next to his chair and the CS Maria sits alongside him facing a computer screen, which is visible to both. She asks him to sit and adjusts his body and head, so he faces the tube and ensures his hands are holding his cheeks, while his feet are flat on the floor. She explains that there will be two tests: one with him breathing normally and a second with rapid force and duration. At this point, the technician stands up and puts a mask on her face to demonstrate the forceful breathing, showing her chest deflating as she blows out the air. There is a calm air as the first test is undertaken, with Jose breathing into the tube and Maria talking calmly through the process saying, “breathe normally”. Both are now intently watching the animated image of a pair of lungs rising and falling on the screen and the red line of the measurements tracking in a looping linear pattern across the screen. At the start of the second test, Maria stands right next to Jose and using a raised voice says “*encher rapido*!” (breathe in quickly!). Her body mimics breathing in rapidly and then blowing air out, saying “*aguenta, aguenta*” (hold hold hold it) before changing the tone of her voice to the point when he can stop saying calmly “*respirar normal*.” The embodied act of breathing and forcing the air out is played out to both in real time on the screen. Maria checks the reading and asks Jose to try again as it could be better, to see, she adds, if he can “improve”. Throughout the lung function test, there is consistent feedback from Maria about his performance—“*estas a melhorar*,” (‘you’re getting better’) and there is a smile of semi satisfaction from Jose when Maria announces that the last one was the best. She tells him this result will be sent as a written report within a few weeks. This report is something that, for Jose, as it is for most participants, eagerly awaited and valued even if it is only, as Jose and others I met frequently stated, to confirm everything is normal.

As this brief excerpt illustrates, anthropometric measurements such as BIA and lung function testing are co‐produced, the product of significant ‘interembodied’ (Bunkley 2022) labour and ‘ontological choreography’ (Cussins 1996) on behalf of the counters and the counted. They also enact and produce numbers that are significantly normed. As a result, the pursuit of health as an individual project of self‐improvement is never totally removed from the relational dynamics surrounding anthropometric measurement, despite the ever‐present potential for objectification and medicalisation. Indeed, with an emphasis on repeated measures, which are often predicated on an assumption of incremental increase in capacity as part of the testing protocol, as is the case in lung function testing, self‐improvement might be seen as built into the technologies themselves.

However, diverse articulations of healthism also emerge in other routine dynamics of cohort clinics and their evaluations. This was especially evident among older birth cohort participants in Brazil and the United Kingdom who have been involved for a much longer part of their adult lives. For these participants, who were in their mid‐30s, reflections on the meaning and significance of the numbers that constituted their measurements were entangled with what was perceived as an ability but also a sense of responsibility to improve their health over time (Crawford 2006). For example, although there was a certain amount of anxiety surrounding anthropometric measurements among the Pelotas participants in Brazil, they also expressed expectations that the current measurements would be better than those at the last visit or, alternatively, a sense of shame and guilt for not having done their part to improve their measurements. This was reflected in the comments of one CS in Pelotas, who observed, “when we are taking measurements, they [participants] ask “oh, have I changed? How was I 10 years ago?” In fact, this kind of participant engagement is often seen as something beneficial, providing an opportunity to instil a mindset of monitoring or self‐care, as one of the Pelotas CS said, “we always encourage them.”

For many other birth cohort participants involved in our project, anthropometric measurements undertaken in the cohort evaluations were shaped by and, in turn, themselves influenced how they monitored their health beyond the confines of the cohort study. Although healthcare in doctor–patient contexts has become an important vector for the promotion of healthism (Turrini 2015), we show how this also extends to include the work of cohort data collection, where screenings and tests necessarily seep into and shape daily experiences. Given such dynamics, a certain self‐consciousness around the dynamics that produce healthism was not uncommon to witness in the evaluations.

A certain striving for perfection was clear to witness for several of the study participants at ALSPAC’s ‘Focus at 30’ clinical evaluations in Bristol. Although ALSPAC’s CS tended to balance encouragement and neutrality in their interactions with participants, there were exceptions, such as encouraging participants not only to complete but also to excel in fitness and strength measures. In this case, the CS expressed his high expectations of Ben, a participant who worked in the army. Although the cardiopulmonary exercise testing (CPET) was underway, the CS watched Ben’s impressive progress and remarked that his army training was responsible for this. As Ben continued cycling, the CS asked, “have you got a target in mind?” By the end of the test, Ben had a very high overall score, and the CS said, “this is what the British army are made of.” CS at ALSPAC tended to attribute impressive results to a participant’s specific attributes and/or lifestyle and depersonalise those casual encouraging assessments of how participants did on physical tests when the results were less impressive.

By contrast, in other more sensitive areas of anthropometric measurement such as weight, CS made every effort to treat bodies and their data neutrally, rooted in their everyday practices of sensitivity and professionalism. Nonetheless, a moral reading of body weight within a healthist framing is common (e.g., Clark 2018). It is also something some cohort participants are aware of and may have internalised, but not always in plain view. As one ALSPAC participant, Katie, who had had bariatric (weight loss) surgery, commented, “people (ALSPAC staff) don’t know what this a symptom of and how that person has worked…they just… see a number.” Decontextualisation in the work of measuring and monitoring bodies in cohort evaluations is, in this sense, not only personal but also structural, highlighting how participants feel the ways in which “an allegedly neutral and desirable goal functions as a moral category allegedly expressive of individual worth” (Mackert and Schorb 2022, 3). In the case of weight measurement in cohort evaluations, it is therefore difficult to separate reductionism and objectification of the body from a moralising emphasis on individual responsibility for health.

For younger cohort participants, such as those in Generation R in Rotterdam (aged 17–20), where participants were much more aware of each other as a community, an extended form of self‐quantification created competition not only with themselves but also with each other. In certain Rotterdam schools, much of a class cohort were, to some degree, aware of one another as fellow cohort participants. Often, participants came to the research centre already knowing what to expect and how they wanted to perform, based on their peers’ previous performances. Gert and his best friend Jurgen were two of these participants who competed not only in their grades or sports but also in how they performed in Generation R. Gert explains: “So there was the grip strength exercise [and] there was a 0.5 kilo difference in his favour, which really ticked me off!” He laughs and continues “[but] There was a jumping exercise, and I outclassed him by about a factor of 1.5! … So it’s always us comparing results and scores”.

In Generation R, what might inadvertently be seen as the ‘gamification’ of health (Maturo 2015, 87) was also used during the clinic visits for certain tests. This included the use of a mobile phone application that monitored participants’ screen time and music volume and provided regular pop‐up questions about their current emotional state. Although such a carefully planned application seemed like a useful way to collect data, many of the participants were irritated by being monitored and by the constant pop‐ups, with one participant likening it to “big brother is watching”. As a result, many deleted the application without completing it. This showed that being quantified and monitored in their daily lives may have its limits for many of the participants.

Although measuring and monitoring biological and physiological aspects of the body might be interpreted as having the potential to objectify participants, our ethnographic work suggests that this process of calculating the body is consistently embedded within, and often relies upon, diverse narratives of healthism, implicating the agency of participants and researchers. This is manifested in the technologies and protocols that are themselves dependent on repeat measurements and incremental improvements such as the lung function tests, alongside the social dynamics of the cohort clinics and evaluations. Here, moral framings and valorisation of ‘good numbers’ by participants and technicians are part of the relational fabric of data collection. Yet, wider social contexts, where values of healthism are more explicit, also seep into and shape these moments of medical and biological measurement. This is evidenced as much in how some participants compare their cohort ‘performance’ with their own personalised health tracking as it is in the gamification of health, where the values of competition and comparison resonate with some, if not all, of the younger cohort participants.

Healthism, although often unstable in the social practices of cohort data collection due to its implicitness, or an absence‐presence evidenced by the coexistence of health neutrality and morality, is therefore also a resource that facilitates participation. For both researchers and participants, healthism mediates the potential for medicalisation but also becomes entangled with practices of quantification, where numbers acquire moral significance. The next section examines how, in the formal and informal circulation of individual participants’ screening and test results, we see a similar ambivalence in the value of healthism, even as this is unevenly and diversely manifested, reflecting the wider socioeconomic contexts and variable limits of public health.

## 5 Taking Tests, Receiving Results

Although the primary purpose of birth cohorts is to examine population‐level development and health outcomes, the results of the tests and examinations that are undertaken can have an impact on individual participants and their families. This includes their awareness, perception and narratives around their own health. Data collection can thereby serve as an implicit exchange; the cohort collects research data and, in return, provides some participants with health information. This can offer reassurance, lead to suggestions and minor lifestyle changes or, on occasion, lead to life‐saving interventions. However, more significantly, the tests and examinations can provide vital healthcare for those with fewer resources to access often inadequate public or private healthcare. Yet, in each of the four cohorts, there were significant variabilities in the sharing of results and in how they were communicated.

In each of the cohorts, there are results of ordinary measurements that, as we have seen in the previous section, are sometimes visible to participants, such as height, weight, waist circumference, grip strength and blood pressure. At the same time, the CS may give offhand feedback for information, encouragement or small talk about, for example, fitness. Yet, there is variation when it comes to whether, when, how and to what extent birth cohort studies formally return individual results of participants’ health examinations and how participants perceive the purpose or importance of those results. How and which results are shared must also be understood in the context of broader public health infrastructures—or lack thereof—which, in turn, also shapes participants’ feelings about results and the cohort studies they are enrolled in. In this sense, how test results are managed within the institutions and infrastructures of birth cohort studies provides a particular window onto the varieties of ambivalence associated with healthism and how the rights and responsibilities of cohort researchers and participants are navigated.

In ALSPAC, although participants can, for example, see numerical results, medical imaging and electrocardiogram (EKG) graphs briefly on the screens during and after testing, it is generally only clinically concerning blood pressure results that are formally shared via a take‐home letter at the end of a clinic visit. If other test results, such as blood lipids, are outside specified ranges, the cohort will notify the health authority, who, in turn, contact the participants for extra medical tests. Nonetheless, although most ALSPAC participants stressed that they understood the difference between cohort participation and receiving healthcare, the clinic visits were also perceived as a kind of health check, even if only a limited or incomplete version of one. They often still believed the study *would* identify anything amiss with their health.

A sense of both acceptance of this reality *and* reassurance that their health was being monitored was evident in an ALSPAC intergenerational interview. As Laurie explained, alongside her daughter:

You see, obviously we were giving them [the cohort research] something [data], but I think we got much more back. Whereas they could have just done the scan and not told you anything[..]they say: ‘we’re going to send this off now, and if they see anything on it, they will contact you,’ but they obviously can’t tell you there and then.

Knowing the results of the cohort’s clinic data collection can result in it being perceived as a health check, therefore making participation valuable and incentivising continued participation. The UK’s cost‐of‐living crisis, coupled with the ongoing effects of multiple government austerity policies, alongside cuts to public services, and a health system that is close to breaking point due to waiting times for referrals (Hunter 2023), can make involvement in a birth cohort study advantageous by providing valuable health information. ALSPAC’s provision of results, although delimited, nonetheless sustains some of the values of healthism by emphasising the extra benefits of identifying potential health risks in the context of a public health crisis.

However, these results could also be far from reassuring and instead provoke certain anxieties, impose new burdens of responsibility and create a sense of moral blame (Turrini 2015). This was evident in how Katie reacted when receiving a high blood pressure referral letter in the clinic. She explained in a follow‐up interview: “When [the CS] said, ‘oh, we’ve got high blood pressure’, my brain was like, well, that’s not a shock because I’m overweight. I guess it just highlighted weight even though that wasn’t even mentioned other than standing on the scales”.

By contrast with ALSPAC, in Generation R in the Netherlands, results for certain tests are consistently shared at the end of the visit, and referral letters can be issued, ranging from eye checks to more expert advice. The compulsory visit to the *uitschrijfarts*, or ‘discharging doctor’, is the closing encounter of the cohort evaluation in Generation R and is intended to share test and scan results that are immediately discernible, such as vision, allergies and hearing. It is also here that participants have a chance to express any health concerns.

Such extensive provision of recommendations is an extra benefit considering Dutch public healthcare is based on the principle and practice of quality care and access for all. Similar to ALSPAC, many Generation R participants saw the examinations as an extra and extensive health check, and one participant, in reference to the magnetic resonance imaging (MRI), compared the clinic visits with the regular car inspections required to ensure compliance on Dutch roads. Therefore, the clinic visits were part of what they considered their special ‘care’ package. At the same time, by contrast with ALSPAC, when referral letters are distributed at the end of a clinic visit, the decision and onus to act are placed purely in the hands of the participants. Such an emphasis on individual self‐determination over health coincides with Dutch healthcare principles, which stress self‐awareness and self‐control (Buijsen 2010).

For some, this concluding visit with the discharging doctor is a pleasant encounter, and participants walk away reassured and secure in their health. For others, the meeting functions as a feedback mechanism for behavioural and lifestyle changes to improve their health, including referrals to doctors or clinics for further examinations. Aisha, aged 17, was one of the participants for whom questions about her health status had been raised at her meeting with the discharging doctor during the last visit, and she expressed her anxiety about the upcoming visit as a result. She nervously touched her right ear while she explained: “I hope [my ear] is still ok, but I don’t know. They also found something in the MRI… something in my brain and they had to do another checkup after but thankfully it was nothing. That was a huge scare for all of us!” The stress was also palpable in Aisha’s mother, Marya, who followed her teenage daughter closely through the various examination rooms. On this occasion, they left with referrals to an optometrist, an additional medical hearing test and a prescription for an EpiPen for a severe allergy. Marya was in disbelief at the extra clinical visits she was facing, which were now her responsibility to follow up.

Although many of the Generation R parents saw the routine clinical evaluations as a hugely beneficial extra check and this was a driving force of motivation for participation, the immediate delivery of results has also led to parental and family stress or even resentment, as the comments above suggest. The giving of comprehensive test results in Generation R means that participants are therefore implicated in an ‘imperative of health’ (Crawford 2006, 403) linked to identifying risks and dangers and the individualised need and responsibility to control them. Instead of providing peace, this can “aggravate the very insecurities they are designed to quell” (Crawford 2006, 415). Nonetheless, although the above case study represents an extreme example, other participants in Generation R received immediate referrals and important lifestyle recommendations, for example, for increasing bone density or improving hearing, or were called later about MRI results that led to exceptional life‐saving discoveries such as heart issues or brain tumours. Receiving test results might therefore trigger a whole spectrum of responses, from a felt sense of control to increased anxiety, yet also extreme gratitude if a serious health anomaly has been identified.

Although for both ALSPAC and Generation R, participants are given presents—discount or gift vouchers that engage the ‘contract’ of exchange in cohort research—this was not the case in Generation 21 in Porto. Although no such incentives or compensation was offered to participants, by contrast, nearly all physiological and anthropometric measurements were returned to participants via a formal report and letter within 3 weeks after the individual cohort evaluation. This mode of giving results, as in other cases outlined above, informed how the values of healthism were articulated in the dynamics between participants and Generation 21 researchers, shaping their experiences of and thoughts about data collection.

In Porto, test results of all the anthropometric measurements, including body mass index, lung function testing, pain tolerance, blood tests and DEXA scans (where applicable), were provided to participants and their parents. As one of the technical staff commented, this was important because ‘we're not giving them anything else in return’. It was a pointed comment that reflected, on the one hand, a widely shared sense that despite never having the financial resources to offer incentives to participants, a significant amount had been achieved in maintaining the cohort, but, on the other hand, a sense of anxiety about how to maintain cohort participation. Porto researchers recognised that in the absence of any other compensation for ongoing participation, detailed health reports from the cohort evaluations were a crucial mechanism for doing this. For Porto cohort participants too, it seemed that these standardised ‘clinically’ presented feedback letters were extremely valuable, often carefully and meticulously saved at home or routinely taken to a general practitioner. The latter action was often conveyed in terms of both an obligation and a responsibility to ‘society’ (*sociedade*) that contributed to the collective effort to ensure the national healthcare system was informed and knew about these records. Here, therefore, although healthism shaped individual acts of health responsibility for cohort participants, this was also done with a view of contributing to a wider greater good.

At the same time, for some Porto cohort members, participation was more than personally adding an extra piece of information to public health records. It was, in fact, a vital component of their healthcare in the context of a failing public health system with insufficient resources (Legido‐Quigley et al. 2016). Although many participants, regardless of their socioeconomic resources, would often state initially, ‘*nao custa nada*’ (it does not cost anything), in response to questions about their ongoing participation in Generation 21, for some it was an essential form of basic care, without which their health would have suffered.

This was the case for Angela, who worked as a cleaner for a local optician, and her 18‐year‐old son Antonio. They lived on the outskirts of Porto, far away from the gentrified centre, where dilapidated rural and urban infrastructures exist side by side. Her employer was shocked to hear that she was part of Generation 21. He thought that it was only for the ‘higher class’, to which Angela replied that it “is for all the babies, there’s no discrimination”, revealing a certain resistance to a narrative of individual benefit. But there was also a sense of luck in how Angela talked about her participation; to her, it was “completely different to going to the doctor… there (at Generation 21) they know everything about you.” With pride and gratitude, she recounted how Antonio had experienced a series of mysterious and frightening allergic reactions and how the cohort study got in contact because of the results they sent. Angela took these results to the doctor to realise that it was a simple allergy that could be treated.

For the Pelotas Birth Cohort Study in Brazil, the results occupied something of a hybrid mode between the other examples already outlined. Some results are given back to participants at the end of their clinic visit, including the DEXA scan and lung function testing, whereas other information such as blood pressure is provided ad hoc during the evaluations. Interviews with the CS revealed how providing some test results was perceived as motivating, but the lack of resources and the inability to provide more information to participants also generated a certain degree of ambivalence. As one researcher put it:

Today they are leaving with the full body composition assessment, with spirometry data. This also helps to keep the participants motivated and prevents them from dropping out, right? But there are things that we can’t do… how am I going to give the results now, of the biochemistry… of blood sugar, cholesterol done when they were age 22? There was perhaps already something there, and the person doesn’t know!

Despite this, participants very much perceived the clinic as providing vital and essential clinical health check‐ups. The possibility of accessing what is understood as ‘holistic’ health information and knowing that parent(s) and child(ren) are being cared for and monitored is considered extremely valuable by participants. As one Pelotas participant stated, “if something is not right, they (CS) will let you know.” In Pelotas, then, perhaps less than receiving individual results, the security provided by being part of the study is enough of a ‘return’. This is especially so in a context where access to public health is uneven and precarious, where participation, for many on low incomes, provides an alternative way to take care of their health (see also Mathers et al. 2025). At the same time, the inability to provide comprehensive information on cohort test results—itself an outcome of fewer resources compared with European‐based cohorts—means that individual responsibility for self‐care continues to be a motivating dynamic in the LMIC setting of Brazil. In Pelotas, remaining in the cohort as an alternative form of care means subscribing to and benefiting from the self‐determination of healthism, ascribed in part through cohort participation, even when conditions for accessing health services are particularly adverse.

We can see from the above comparative analysis that the variation in formal and informal communication of results are a product of institutional cultures, resources and concerns about the balance between motivating participants, over‐medicalising them or increasing their sense of individual responsibility for or anxiety about their health. At the same time, these practices have evolved against a background of differences in public healthcare provision and infrastructures. As a result, in Generation 21 and the Pelotas Birth Cohort Study, the inadequacy of healthcare provision or the inability to provide other rewards or compensation also impacts the context, scope and consequences of sharing personal health information. For participants, the ability to obtain healthcare results through cohort involvement can be a bonus extra, as in the case of Generation R, a stopgap to failing and inadequate healthcare systems in LMIC contexts such as Brazil, but also for some in Generation 21 and, perhaps increasingly so, for ALSPAC participants. As a result, the value of healthism articulated as a morally framed pursuit of health is somewhat conflicted in the context of healthcare information‐sharing in cohort research. On the one hand, just as Crawford (2006) highlights how the variable influences of healthism are linked to social class, we have shown how the specific scope and limitations of public health provision inform the scope and perceived value of test results. At the same time, although the aspects that drive healthism provide a motivation for participation and are a mechanism for delivering benefits to participants, it also has the ever‐present potential to engender a burden of individual responsibility for health that can increase anxiety, stress and even stigma.

## 6 Conclusion

Like other health institutions, from local clinics to national and international health organisations, longitudinal birth cohorts have come to a crossroads in a context where both the ongoing promise and failures of neoliberal market economics continue to unfold. This shapes the evolving challenges for birth cohorts centred on attrition—loss of participants to the study—funding, navigating changing research and public health priorities and the newly emerging possibilities for capturing social and biological data from individuals and communities. The close entanglement between the origin, purpose and aim of cohort studies and the message of health as a pan value—so central to healthism—are key components of these challenges. If promoting health enables people to control factors that would otherwise lie outside their control, there is paradoxically an implicit acknowledgement that the onus of responsibility lies outside the individual, but only up to a certain point. How and when does a factor become individually controllable? Who decides this and to what end? Although we argue that health institutions, and especially longitudinal population studies such as birth cohorts, are at a crossroads in 2025, such a paradoxical state of being is familiar territory. Crawford's crystallisation of these complex tendencies within the concept of healthism points to the entanglement of cultural norms, internalised stigma, relationality and practices of giving and capturing biological data.

In this article, we have aimed to recontextualise Crawford's original framing by showing the diverse social dynamics through which healthism operates in the context of a relatively under‐examined terrain of healthcare research, the longitudinal birth cohort study. In this sense, our analysis, rather than focusing on one disease condition or community, provides a unique contribution to understanding how healthism emerges, evolves and is made relevant within a particular health institution. Although the sociohistorical context of individual birth cohort studies is varied, we have highlighted how a focus on improving population health has been central to the founding principles, histories and aims of birth cohort studies as a ‘tool’ for intervening in public health. At the same time, we have explored how birth cohort research is evolving, and in dialogue with, the diverse values and articulations of healthism. By adopting a comparative approach, we have pointed to the sociohistorical specificity of individual birth cohort studies and how their emergence, ongoing success and continued relevance are enmeshed with the contemporary histories, values and inadequacies of public healthcare provision.

By providing an ethnographic perspective on the work of data collection, we have first sought to demonstrate how healthism is consistently entangled with the ever‐present possibility of objectification, reductionism and medicalisation in the data collection work of birth cohort studies. Measuring and monitoring the body and the biological are often achieved, or made possible, through recourse to the moral value of individual actions to seek or achieve health in the relational dynamics of the cohort evaluations. Such moral framings can also be part of the ‘technology’ of measurement itself, with an emphasis on incremental improvement and repeated measures. At the same time, the effort to resist a healthism discourse to retain the ‘neutrality’ of numbers in cohort data collection can also result in a felt sense of decontextualisation that misses the complexity of participants’ lived lives. Still, aspects of healthism can seep into cohort data collection practices from the outside by intersecting with the self‐monitoring that is already part of participants’ daily lives, in part through their own efforts to optimise their health or the gamification of health/lives among younger birth cohort members.

While demonstrating the consistent dependency on and presence of healthism in the work of cohort data collection practice, we also show how this is an ambivalent element of birth cohort research, revealed in how results are communicated to participants. Our comparison across the four cohorts points to significant variability in how results are shared, spanning a spectrum from virtually full clinical reports to little or no formal information given, aside from concerning results. We argue that this variability, and the critical reflections by the participants and cohort research personnel, articulates a tension that centres on questions of rights and responsibility. This includes the responsibility of the cohort to its participants in a context of partial or inadequate public health or the responsibility to avoid over‐medicalising participants’ lives and inducing anxiety. Equally, it emphasises participants’ rights to information that is individually perceived as beneficial, useful or valuable for their health and which, despite its often limited actual scope, provides a powerful motivation for ongoing participation in the birth cohort.

In summary, healthism is therefore embedded in the work of birth cohort research, but it is an ambivalent and multivalent aspect of this terrain, an uneasy but necessary element of the work of collaboration between the cohort research community and cohort participants. Clearer recognition of how healthism is entangled with the dynamics of birth cohort studies is important for understanding this under‐examined institutional context of health research and a necessary first step in efforts to rethink the situated and relational stakes of lifelong research participation.

## Author Contributions

**Sahra Gibbon:** conceptualization, methodology, formal analysis, data curation, writing – original draft, review and editing. **Carola Tize:** formal analysis, data curation, writing – original draft, review and editing. **Taylor Riley:** formal analysis, data curation, writing – original draft. **Tatiane Muniz:** formal analysis, data curation, writing – original draft.

## Ethics Statement

Research permission was granted for the entire study by University College London Research Ethics (ID: 0547/006), and local approval was sought and secured within the four individual cohorts and their affiliated university institutions.

## Consent

Informed consent was obtained from all subjects involved in the study.

## Conflicts of Interest

The authors declare no conflicts of interest.

## Permission to Reproduce Material From Other Sources

The authors have nothing to report.

## Data Availability

Most of the data that support the findings of this study are available from the corresponding author upon reasonable request. Because of privacy reasons, not all data may be made available.

## Endnotes

^1^
Including, for example, blood, hair, placentas and teeth.

^2^
The variability in numbers between cohorts was dependent on data collection times and logistical challenges to follow up.

^3^
Interviews were conducted with the data collection staff and cohort study principal investigators (PIs).

^4^
Including Generation R, Erasmus Medical Center; Generation 21, University of Porto, ISPUP; the Pelotas Birth Cohort Study, Federal University of Pelotas; and the ALSPAC Law and Ethics Committee (Ref.: B3613), University of Bristol.

^5^
In line with the principles of each cohort, incentives or compensation for participation in the form of gift certificates were determined by ALSPAC, Generation R and Pelotas. By contrast, Generation 21 did not permit such rewards.

^6^
Generation R works in close collaboration with the School of Law and Faculty of Social Sciences of Erasmus University Rotterdam; the Municipal Health Service Rotterdam area; the Rotterdam Homecare Foundation, Rotterdam; and the Stichting Trombosedienst en Artsenlaboratorium Rijnmond (STAR‐MDC), Rotterdam.

^7^
The Wellcome Trust (Grant Ref.: 217065/Z/19/Z) and the University of Bristol provide support for ALSPAC. This publication is the work of the authors, who are guarantors for its contents. We are grateful to the ALSPAC participants and families and the wider scientific team, including midwives, interviewers, computer and laboratory technicians, clerical workers, research scientists, volunteers, managers, receptionists and nurses. The study website contains details of all available data through the data dictionary and variable search tool: http://www.bristol.ac.uk/alspac/researchers/our‐data/. A comprehensive list of funding is also available at http://www.bristol.ac.uk/alspac/external/documents/grant‐acknowledgements.pdf.

^8^
14,203 mothers were initially enrolled, and subsequently, 14,833 were enrolled as of September 2021. With additional recruitment, the total sample size for data collected after the age of seven is 15,447 pregnancies, resulting in 15,658 foetuses, with 14,901 children alive at 1 year of age. A total of 12,113 of the mothers' partners have been in contact with the study. Of the original parent partners, 3807 are currently enrolled (see Boyd et al. 2013; Fraser et al. 2013; Northstone et al. 2019).

^9^
‘Clinical Staff’ refers to data collectors undertaking routine measurements and monitoring in the 4 cohort studies. Although in ALSPAC and Generation R, they were referred to as clinical staff, in Generation 21 and in the Pelotas Birth Cohort Study they were variously referred to as the researcher, interviewer, technician or practitioner.
